# Statin treatment prevents the development of pulmonary arterial hypertension in a nonhuman primate model of HIV-associated PAH

**DOI:** 10.1038/s41598-019-55301-9

**Published:** 2019-12-27

**Authors:** Whitney Rabacal, Finja Schweitzer, Emily Rayens, Rebecca Tarantelli, Patrick Whang, Viviana Cobos Jimenez, Judy A. Outwater, Karen A. Norris

**Affiliations:** Center for Vaccines and Immunology, University of Georgia, Athens, 30602 Georgia

**Keywords:** Inflammation, Hypertension, Interventional cardiology

## Abstract

Pulmonary arterial hypertension (PAH) is a life-threatening disease characterized by pulmonary vascular remodeling, elevated pulmonary arterial pressure, and right heart failure. Human immunodeficiency virus (HIV)-infected individuals have a higher incidence of PAH than the non-HIV infected population and evidence suggests a role for systemic and pulmonary inflammation in the pathogenesis of HIV-associated PAH. Due to their pleiotropic effects, including immune-modulatory and anti-inflammatory effects, 3-hydroxy-3-methylglutaryl coenzyme A (HMG-CoA) reductase inhibitors (statins) have been considered for the treatment of PAH, with conflicting results. The effects of statins on HIV-associated PAH have not been specifically evaluated. We have developed a non-human primate (NHP) model of HIV-associated PAH that closely mimics HIV-PAH using simian immunodeficiency virus (SIV)-infected rhesus macaques (*Macaca mulatta*). We determined that treatment of healthy macaques with atorvastatin prior to and throughout SIV infection prevented the development of SIV-associated PAH. Additionally, SIV-infected macaques that initiated atorvastatin treatment during the early chronic disease stage had reduced incidence of PAH compared to untreated animals. Statin treatment reduced inflammatory mediators TGF-β, MIP-1α, and TNF-α and the numbers of CD14^dim^CD16^+^ non-classical monocytes, and CD14^+^CCR7^−^CD163^−^CD206^+^ alveolar macrophages previously shown to be associated with SIV-PAH. These results support the concept that statins reduce inflammatory processes that contribute to PAH and may provide a safe and effective prophylactic strategy for the prevention of PAH in HIV-infected individuals.

## Introduction

Pulmonary arterial hypertension (PAH) is a subgroup of pulmonary hypertension that includes idiopathic PAH and heritable forms, as well as PAH associated with congenital heart disease, connective tissue disease, portal hypertension, human immunodeficiency virus (HIV) and other infections^1^. The hemodynamic definition of PAH is defined by a mean pulmonary artery pressure (mPAP) at rest ≥25 mmHg with a pulmonary capillary wedge pressure <15 mmHg^2^. PAH occurs in approximately 0.5% of HIV-infected persons, which is 100 to 1000 times greater than the prevalence of PAH in non-HIV infected populations^3^. Despite the improvements in HIV-associated morbidity and mortality, the prevalence of PAH has not changed significantly in the post-ART era^3,4^. Recent echocardiaographic studies of HIV-infected outpatients found that between 15% and 35% had elevated pulmonary artery systolic pressures^5,6^, indicating that PAH may be even more common than previously thought. Even with diagnosis and treatment, prognosis remains poor for both HIV and non-HIV-associated PAH.

3-hydroxy-3-methylgluaryl coenzyme A (HMG-CoA) reductase inhibitors (statins) can suppress inflammation^7^ independently of their lipid lowering effects^8,9^. Statins have markedly improved morbidity and mortality in clinical trials of disease^10,11^ and transplantation^12,13^, and ameliorated disease in experimental models of autoimmunity^14,15^. Through their pleiotropic functions, statins are hypothesized to mitigate PAH pathogenesis by maintaining vascular cell homeostasis and preventing inflammatory feedback cascades that promote aberrant proliferation and vessel occlusion^9,16,17^. Statins have been shown to suppress vascular inflammation, inhibit pulmonary smooth muscle cell proliferation, and improve hemodynamic parameters in experimental rodent models of PAH^18–21^; although, limited clinical trials in late stage PAH have yielded conflicting results^22–27^. Several studies have reported moderate improvement in PAH-associated biomarkers but long term physiologic benefits were not generally evident^26–28^. An examination of statin efficacy in preventing or treating HIV-associated PAH has not been tested, and it is unclear whether the results of previous statin trials are applicable to HIV-associated PAH populations, where chronic immune activation and inflammation are believed to play key roles in the development of cardiopulmonary co-morbidities.

Until recently, a lack of adequate animal models that faithfully recapitulates the immunologic, histologic, and hemodynamic features of human PAH has inhibited understanding of the disease pathobiology, and hindered the development of novel therapeutic strategies. Rodent models of PAH have provided key insights into PAH pathogenesis but do not mimic the complex immunological dysregulation that contributes to idiopathic^29,30^, autoimmune^31^, and HIV-PAH^32,33^. Rhesus macaques have been extensively used for modeling HIV infection and preclinical testing of therapeutics^34,35^, and closely mimic human hemodynamics during healthy and PAH diseased states^36^. Moreover, SIV-nfected macaques develop pulmonary arterial lesions, similar to patients with idiopathic PAH, characterized by intimal and medial thickening with luminal occlusion^37–40^.

Through longitudinal evaluation of SIV-infected macaques by right heart catheterization (RHC), Tarentelli et. al, recently established a nonhuman primate (NHP) model of HIV-PAH where 52.4% of SIV-infected macaques develop elevated pulmonary pressures (mPAP ≥ 25 mmHg) within 6–12 months following SIV infection^36^. SIV-mediated PAH in these animals was associated with increased levels of pro-inflammatory cytokines (TGF-β, MIP-1α, and TNF-α) and an increased frequency of pro-inflammatory monocytes and pro-fibrotic macrophages^36,41^. Interestingly, SIV-infected NHPs that did not develop PAH had increased levels of plasma IL-15 and lung tissue IL-10^36,41^. In the present study, we tested the effects of statin treatment on the development of PAH and associated immunologic phenotypes in SIV-infected rhesus macaques.

## Results

### Early statin treatment prevents PAH in SIV-infected NHPs

**Figure 1:**
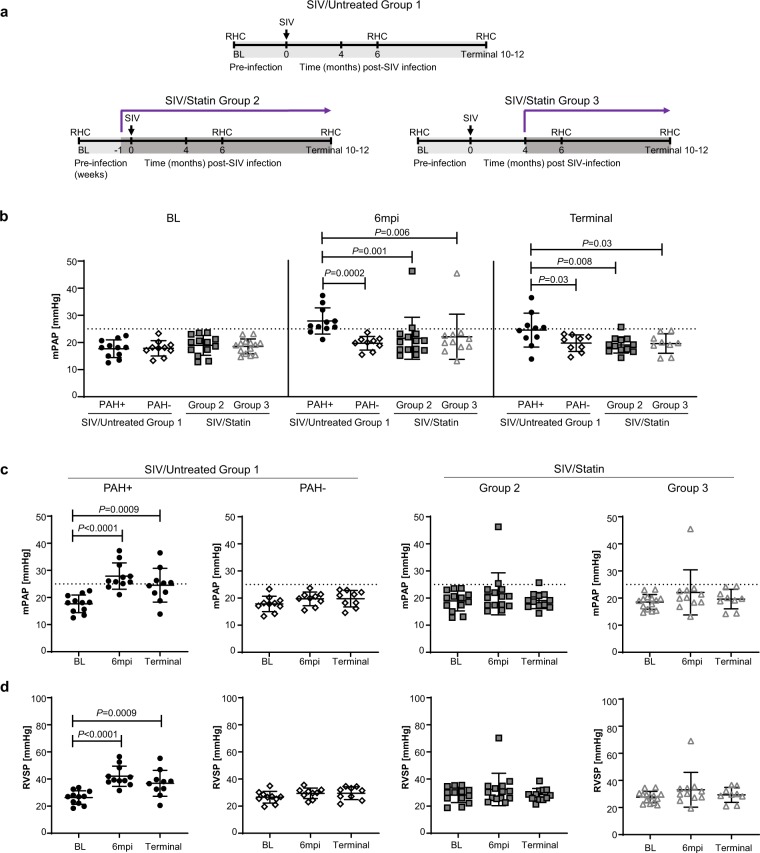
Hemodynamic evaluation of pulmonary arterial hypertension with statin treatment in a NHP model of HIV-PAH. (**a**) Study schematic of statin treatment, SIV infection, and serial right heart catheterization (RHC). (**b**) Mean arterial pressure (mPAP), comparing treatment cohorts at baseline (BL), 6 months post-infection (6mpi), and terminal timepoints (10–12mpi). (**c**) mPAP is calculated from (**d**) right ventricular systolic pressure (RVSP), where $mPAP\,(in\,mmHg)=0.65\times (RVSP)+0.55$. PAH is defined as mPAP ≥ 25 mmHg as indicated by the dashed lines. (**b**–**d**) Serial and group characterizations of hemodynamics were analyzed using repeated measures mixed modeling. Data represents the mean ± SD.

52.4% (11 of 21) of SIV-infected macaques developed elevated pulmonary pressures within 6–12 months of infection (SIV/Untreated Group 1 PAH+); whereas 47.6% (10 of 21) maintained normal hemodynamic parameters throughout the course of infection (up to 12 months post-infection) (SIV/Untreated Group 1 PAH−) (Table 1; Fig. 1; Extended Data Fig. 1). To determine if statin therapy in healthy macaques could alter the incidence or progression of SIV-PAH, atorvastatin treatment was initiated in a cohort of NHPs 1 week prior to SIV infection (Fig. 1a, SIV/Statin Group 2; Extended Data Fig. 1) and maintained on drug throughout infection. Hemodynamics were measured by serial right heart catheterizations at baseline (BL), 6 months post-infection (6 mpi), and at terminal (end of study, 10–12 mpi). We were unable to obtain complete hemodynamic data from two animals (monkey 22–16 and terminal timepoint from monkey 47–16). In contrast to untreated controls (SIV/Untreated Group 1, 52.4% PAH+), 14.3% (Table 1, 2 of 14, *P* = 0.03) of statin-treated animals (SIV/Statin Group 2) developed elevated mPAP (monkey 33–16 at 25.4 mmHg and monkey 24–16 at 46.3 mmHg) at 6 months post-infection (Fig. 1). Of these animals, one maintained slightly elevated mPAP until the end of the study (monkey 33–16, terminal mPAP = 25.7 mmHg). At study termination, mPAP of monkey 24–16 decreased to 21.2 mmHg.

**Table 1:** Association between statin treatment and PAH in SIV-infected macaques.

Cohort	SIV/Untreated Group 1	SIV/Statin Group 2	SIV/Statin Group 3
All animals	n = 21	n = 14	n = 12
Male, sex, N (%)	11 (52.4)	8 (57.1)	6 (50)
Age at infection, years (Mean ± SD)	6.1 ± 1.0	8.1 ± 1.9	8.8 ± 1.2
**Primary Outcome**			
Incidence PAH ≥ 25 mmHg 6–12 mpi	11	2	1
Prevalence of PAH (% PAH+)	52.4	14.3	8.3
**Relative Risk of PAH with statin treatment**			
Value	—	0.273	0.159
95% CI, Koopman asymptotic score	—	0.073–0.859	0.028–0.739
**Association between statins and PAH**			
Fisher’s exact test *P* value	—	*0.03	*0.02

CI, confidence interval; PAH, pulmonary arterial hypertension.

*P < 0.05.

We further tested whether statin treatment initiated following SIV infection could prevent the development of PAH or alter progression (Fig. 1, SIV/Statin Group 3; Extended Data Fig. 1). In this group, atorvastatin was administered four months following SIV infection and maintained throughout infection. We were unable to obtain RHC data from 44–16 at 6mpi; terminal RHC data from 26–16, 27–16, and 29–16; and 6mpi and terminal data from 49–16. One animal (31–16) developed rapidly progressing SIV/AIDS and was removed from study at 13 weeks post-infection. Of the 12 remaining monkeys that had hemodynamic measurements following infection, 1 of 12 monkeys (Tables 1, 8.3%, *P* = 0.02) developed PAH (animal 29–16; 6mpi mPAP = 45.5) at 6 months post-infection. This monkey (29–16) had a relatively high baseline mPAP (23.0 mmHg) and was euthanized at 36 weeks post-infection due to *Pneumocystis* pneumonia without performing a terminal RHC. These results demonstrate that SIV-PAH can be prevented through prophylactic and therapeutic intervention with atorvastatin.

### Effects of statins on peripheral CD4+ T cells and SIV infection

**Figure 2:**
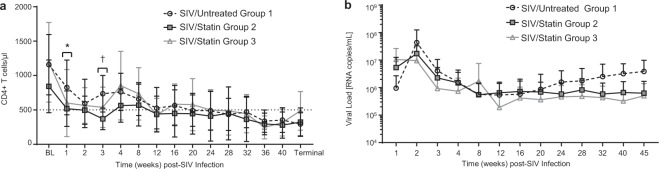
Evaluation of peripheral blood CD4+ T cells and viral load throughout SIV infection and statin treatment. (**a**) CD4+ T cells of SIV-infected macaques comparing SIV/Untreated Group 1 vs SIV/Statin Group 2 or SIV/Statin Group 3. (**b**) Viral load. Data represents the mean ± SD. Differences in CD4+ T cells and viral load were analyzed by repeated measures mixed modeling. CD4+ T cells statistically differed between SIV/Untreated Group 1 vs SIV/Statin Group 2 at 1 week post-infection (*P = 0.02) and 3 weeks post-infection (^†^*P* = 0.0003).

Statins have been reported to inhibit lymphocyte migration which in turn can impair T effector responses necessary for pathogen clearance and viremic control^42,43^. We therefore examined peripheral blood CD4+ T cell levels and plasma viral load throughout SIV infection. CD4+ T cell frequencies in both statin-treated cohorts were similar to SIV/Untreated controls, except in SIV/Statin Group 2 at 1 and 3 weeks post-infection (Fig. 2a); however, cell numbers recovered by 8 weeks post-infection and remained similar throughout the remainder of the experiment. In addition, statin treatment did not significantly alter viral load compared to SIV/Untreated Group 1 controls (Fig. 2b). All SIV-infected macaques displayed the typical decline in peripheral blood CD4+ T cells and a characteristic chronic-phase plasma viral level. Together, these data indicate that statin treatment did not significantly alter peripheral CD4+ T cell homeostasis or viral load in this study.

### Statin treatment prevents alterations in cytokine profiles associated with SIV-PAH

**Figure 3:**
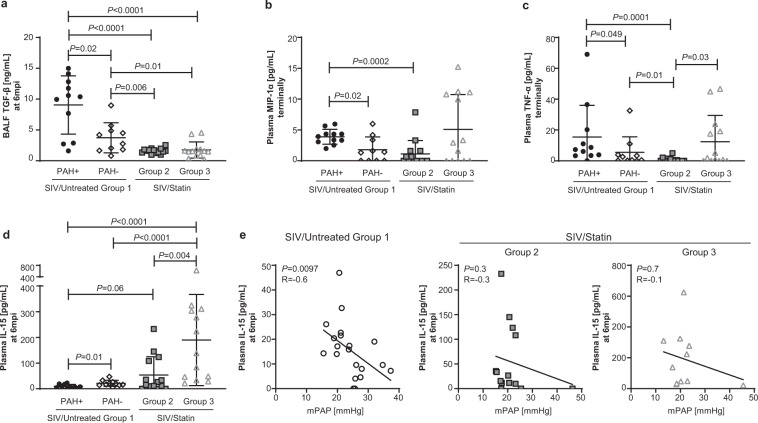
Effect of statins on SIV-PAH-associated cytokine profiles. (**a**) Bronchoalveolar lavage fluid (BALF) TGF-β levels at 6mpi. (**b**,**c**) Terminal plasma levels of MIP-1α and TNF-α, respectively. (**d**) Plasma IL-15 levels at 6mpi. (**a**–**d**) Mann-Whitney U test was used for statistical analysis. Data represents the mean ± SD. (**e**) Spearman correlation analysis between mPAP and plasma IL-15 at 6mpi in SIV/Untreated animals (left), and statin-treated cohorts (center, right); R, Spearman coefficient.

HIV induces a state of chronic inflammation that may drive PAH pathogenesis. Among inflammatory cytokines associated with SIV-PAH, bronchoalveolar lavage fluid (BALF) TGF-β (Fig. 3a, *P* = 0.02) and plasma MIP-1α (Fig. 3b, *P* = 0.02) and TNF-α (Fig. 3c, *P* = 0.049) levels are significantly higher in SIV-PAH+ animals compared with SIV-PAH− controls. To determine if statin treatment could modify these inflammatory mediators associated with SIV-PAH, we compared cytokine profiles in our statin-treated cohorts. Consistent with our hypothesis that statin treatment can suppress SIV-PAH-associated inflammation, levels of BALF TGF-β at 6 months post-infection were significantly lower in both statin-treated cohorts compared to SIV-PAH+ (Fig. 3a; SIV/Statin Group 2, *P* < 0.0001; SIV/Statin Group 3, *P* < 0.0001) and SIV-PAH− controls (Fig. 3a; SIV/Statin Group 2, *P* = 0.006; SIV/Statin Group 3, *P* = 0.01). In addition, terminal level of plasma MIP-1α (Fig. 3b, *P* = 0.0002) and TNF-α (Fig. 2c, *P* = 0.0001) was significantly lower in SIV/Statin Group 2 compared with SIV-PAH+ controls. However, terminal plasma levels of MIP-1α and TNF-α were not significantly reduced in SIV/Statin Group 3, indicating that these responses may not be suppressed when statin treatment is initiated during the post-acute phase of infection.

In contrast with inflammatory cytokines TGF-β, MIP-1α, and TNF-α, IL-15 is elevated in animals resistant to SIV-PAH (Fig. 3d, *P* = 0.01) and is inversely correlated with increased pulmonary pressures at 6 months post-infection (Fig. 3e; left panel, *P* = 0.0097). Plasma IL-15 was elevated in both statin cohorts compared with SIV-PAH+ controls (Fig. 3d; SIV/Statin Group 2, *P* = 0.06; SIV/Statin Group 3, *P* < 0.0001). Moreover, IL-15 levels in both statin cohorts did not correlate with increased pulmonary pressures (Fig. 3e; center, right panels). Collectively, these data indicate that statin treatment suppresses chronic inflammation and may promote cytokine responses associated with SIV-PAH resistance.

### Statin treatment prevents monocyte and macrophage skewing associated with inflammation and fibrosis in SIV-PAH

**Figure 4:**
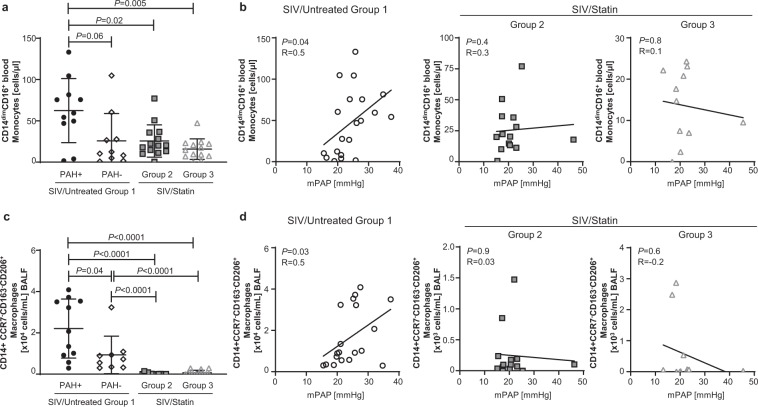
Effect of statins on SIV-PAH-associated monocyte and macrophage phenotypes. (**a**) Absolute cell numbers of CD14^dim^CD16^+^ non-classical monocytes in the peripheral blood at 6mpi. (**b**) Correlation analysis between mPAP and CD14^dim^CD16^+^ non-classical monocytes in SIV/Untreated animals (left), and statin-treated cohorts (center, right). (**c**) Absolute cell numbers of CD14^+^CCR7^−^CD163^−^CD206^+^ macrophages in the BALF at 6mpi. (**d**) Correlation analysis between mPAP and CD14^+^CCR7^−^CD163^−^CD206^+^ BALF macrophages in SIV/Untreated animals (left), and statin-treated cohorts (center, right). (**a**,**c**) Mann-Whitney U test was used for statistical analysis. Data represents the mean ± SD. (**b**,**d**) Spearman correlation analysis; R, Spearman coefficient.

Among cytokine signatures associated with SIV-PAH, the inflammatory mediators MIP-1α, TNF-α, and TGF-β have been previously associated with macrophage populations that promote fibrosis^36,41^. At 6 months post-infection, SIV-PAH+ animals had higher numbers of peripheral blood CD14^dim^CD16^+^ non-classical monocytes (Fig. 4a, *P* = 0.06) and CD14^+^CCR7^−^CD163^−^CD206^+^ BALF macrophages (Fig. 4c, *P* = 0.04) compared to SIV-PAH− controls. Moreover, increased numbers of CD14^dim^CD16^+^ non-classical monocytes (Fig. 4b, left panel, *P* = 0.04) and CD14^+^CCR7^−^CD163^−^CD206^+^ macrophages (Fig. 4d, left panel, *P* = 0.03) correlated with increased pulmonary pressures in SIV/Untreated Group 1 controls at 6 months post-infection. Given the pleotropic effects of statins upon monocyte and macrophage skewing and cytokine secretion^15,44,45^, we hypothesized that statins may dampen myeloid phenotypes associated with SIV-PAH. Consistent with our hypothesis, the number of CD14^dim^CD16^+^ non-classical monocytes were significantly lower in both statin-treated cohorts compared to SIV-PAH+ controls (Fig. 4a; SIV/Statin Group 2, *P* = 0.02; SIV/Statin Group 3, *P* = 0.005) and did not correlate with increased pulmonary pressures (Fig. 4b, center, right panels). Moreover, the numbers of BALF CD14^+^CCR7^−^CD163^−^CD206^+^ BALF macrophages were significantly reduced with statin treatment (Fig. 4c; SIV/Statin Group 2, *P* < 0.0001; SIV/Statin Group 3, *P *< 0.0001) compared to both SIV-PAH+ and SIV-PAH− controls. Furthermore, these macrophage numbers did not correlate with increased pulmonary pressures (Fig. 4d, center, right panels). These data suggest that statin treatment reduces monocyte and macrophage phenotypic skewing associated with SIV-PAH.

### Statin treatment prevents SIV-PAH-associated fibrosis in the heart and pulmonary arteries

**Figure 5:**
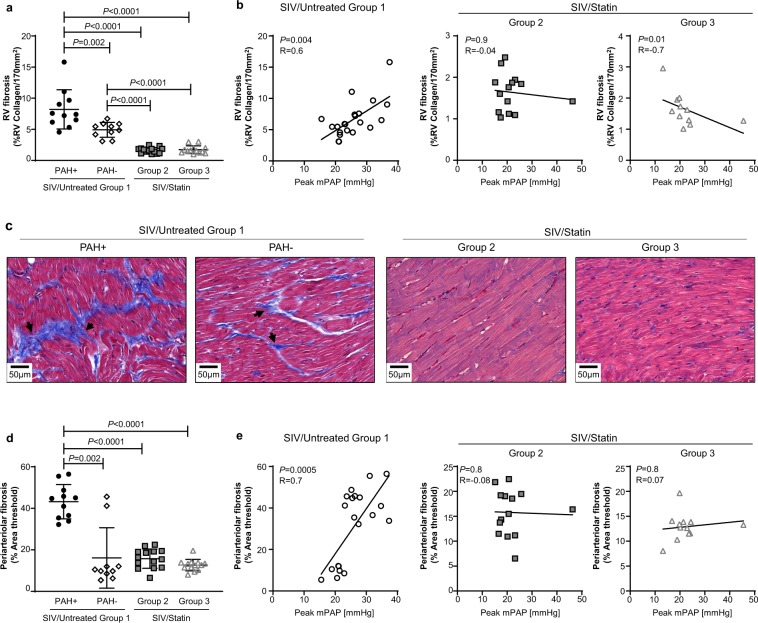
Effect of statins on SIV-associated fibrosis in the heart and pulmonary arteries. (**a**) Quantification of right ventricle (RV) collagen deposition quantified from Masson’s trichrome-stained heart sections. (**b**) Correlation analysis between peak mPAP and RV fibrosis (%RV Collagen/170 mm^2^) in SIV/Untreated animals (left), and statin-treated cohorts (center, right). (**c**) Representative images of Masson’s trichrome-stained right ventricle sections. (**d**) Quantification of pulmonary periarteriolar collagen deposition quantified from Picro-Sirius Red-stained lung sections. (**e**) Correlation analysis between peak mPAP and periarteriolar fibrosis (%Area threshold) in SIV/Untreated animals (left), and statin-treated cohorts (center, right). (**a**,**d**) Mann-Whitney U test was used for statistical analysis. Data represents the mean ± SD. (**b**,**e**) Spearman correlation analysis; R, Spearman coefficient.

SIV-infected macaques exhibit pulmonary arterial lesions similar to idiopathic human PAH; however, the extent of vascular remodeling is relatively mild compared to HIV-PAH^37–40^. Our previous studies indicate that right ventricular (RV) fibrosis is a relatively early manifestation of SIV-PAH and occurs in advance of significant pulmonary vascular lesions^36^. SIV-PAH+ animals had significantly higher levels of fibrosis within the right ventricle (Fig. 5a, *P* = 0.002) and pulmonary arteries (Fig. 5d, *P* = 0.002) compared with SIV-PAH− animals. Collagen levels within both the right ventricle (Fig. 5b, *P* = 0.004; Fig. 5c) and small pulmonary arteries (Fig. 5e, *P* = 0.0005) correlated with increased pulmonary pressures in SIV/Untreated Group 1 controls. Given the substantial dampening of pro-fibrotic immune phenotypes that we observed with statin treatment (Figs. 3–4), we examined RV and lung periarteriolar collagen deposition following statin treatment. Within the right ventricle, collagen deposition was significantly lower in both statin-treated cohorts compared to SIV-PAH+ animals (Fig. 5a; SIV/Statin Group 2, *P *< 0.0001; SIV/Statin Group 3, *P *< 0.0001), and did not correlate with increased peak pulmonary pressures (Fig. 5b, center and right panels). Similarly, within the lung, periarterial collagen deposition was significantly lower in both statin-treated cohorts compared to SIV-PAH+ controls (Fig. 5d; SIV/Statin Group 2, *P *< 0.0001; SIV/Statin Group 3, *P *< 0.0001). Interestingly, statin treatment reduced fibrosis in both the RV and lung to levels lower than those observed in SIV-PAH− controls. These data reveal that statin intervention therapy significantly reduces fibrosis in the heart and pulmonary arteries during SIV infection.

## Discussion

In this study, we evaluated the efficacy of statins on preventing PAH in a rhesus macaque model of HIV-associated PAH through longitudinal hemodynamic measurements and analysis of inflammatory signatures. We determined that treatment of healthy macaques with atorvastatin initiated prior to SIV infection lowered the prevalence of SIV-PAH to 14.3% in treated macaques (SIV/Statin Group 2; 2 of 14) compared to 52.4% among untreated controls (SIV/Untreated Group 1; 11 of 21). Statin treatment prevented SIV-PAH-associated increases in inflammatory cytokines TGF-β, and MIP-1α, and TNF-α, and prevented increases in CD14^dim^CD16^+^ non-classical monocytes, and CD14^+^CCR7^−^CD163^−^CD206^+^ BALF macrophages. Additionally, SIV-infected macaques treated in the post-acute phase were similarly protected from developing SIV-PAH (SIV/Statin Group 3; 8.3%, 1 of 12). Although statins have known anti-inflammatory effects, treatment of infected animals did not significantly alter peripheral CD4+ T cells or viremic control. These results present an optimal therapeutic balance whereby SIV-PAH is prevented by curbing inflammation, without significantly altering viremic control.

As an extension of our investigation into the immunomodulatory effects of statins, we also examined IL-15 levels in statin-treated cohorts. Although primarily associated with maintaining lymphocyte homeostasis and chronic inflammation^46,47^, IL-15 has been recently proposed as a potential anti-fibrotic agent in mouse models of interstitial pulmonary fibrosis and pancreatic fibrosis^48–50^. Likewise, SIV-infected macaques that do not develop PAH have higher plasma IL-15 levels than their SIV-PAH+ counterparts^36^. In this study, statin treatment significantly increased levels of plasma IL-15 and correlated with a decreased incidence of SIV-PAH. Interestingly, the three statin-treated animals that developed SIV-PAH had the lowest levels of IL-15 within their respective treatment cohorts, suggesting a potential biomarker for HIV-PAH risk. Further investigation is ongoing to determine whether IL-15 pathways can be manipulated for the development of novel prophylactic or therapeutic strategies.

In addition to investigating immune signatures, we examined collagen deposition in the right heart and pulmonary vasculature to determine if statin treatment can reduce fibrosis. SIV-PAH is associated with increased right heart and lung periarteriolar collagen deposition^36^. Here we report that statin treatment reduced collagen deposition in both the right heart and pulmonary vasculature compared to SIV-PAH+ controls. Interestingly, statin treatment reduced RV collagen to levels lower than those observed even in SIV-PAH− animals. These data support the concept that statins prevent elevated pulmonary pressures by preventing SIV-associated fibrosis.

Several factors have been proposed that may contribute to the higher prevalence of PAH among HIV-infected individuals, including intravenous drug use, cardiovascular comorbidities, and co-infection with respiratory pathogens^1^. *Pneumocystis* infection in SIV-infected rhesus macaques can lead to life threatening pneumonia (PCP)^51^. In a murine model, Swain *et al*. reported that CD4-depleted animals subsequently infected with *Pneumocystis* developed PAH^52^. In the current study, two animals (29–16 and 46–16) developed PCP. Of these, 29–16 of developed PAH (peak mPAP = 45.5) and was euthanized at 36 weeks post-infection due to clinically advanced PCP; however, we have not observed a correlation between *Pneumocystis* infectionand the development of SIV-PAH (Norris, unpublished). We did not observe other opportunistic infections or comorbidities (neurologic symptoms, systemic hypertension, malignancies) among cohorts throughout this study.

This study builds upon previous work in several ways. This study is the first to demonstrate that SIV-PAH is a preventable disease that can be abrogated through pharmaceutical intervention up to the early chronic phase of infection and possibly later. In addition, these data demonstrate that SIV-PAH pathogenesis is driven by key immunologic processes that include specific inflammatory pathways and pro-fibrotic myeloid populations. Moreover, these processes can be curtailed through preventive therapy using one of the most widely prescribed classes of drugs available, statins. These observations can be potentially extended and applied to both HIV-infected and non-infected individuals at risk of developing PAH.

The full breadth of pathogenic mechanisms driving HIV-PAH is still unknown. Further investigation is necessary to identify novel immune-mediated pathways that may drive or prevent HIV-PAH pathogenesis. Nevertheless, statin treatment successfully inhibited several PAH-associated immunologic parameters previously identified. There are limitations to this study. We did not address whether statin-based therapeutic strategies can ameliorate disease following established PAH, as has been investigated in several clinical trials. We also did not evaluate the effect of antiretroviral therapy (ART) on development of SIV-PAH or whether statins are effective in combination with ART.

This prospective study is the first to demonstrate the efficacy of statin prevention therapy in a highly relevant pre-clinical, NHP model of HIV-PAH, and identify potential immunologic biomarkers of disease progression that are affected by statin treatment. Herein these data demonstrate the efficacy of statin therapy in the absence of confounding factors such as illicit drug use, ART, and non-*Pneumocystis* co-infection. These data are clinically significant because they suggest that HIV-PAH can be prevented early in HIV infection by administering a drug that is already FDA approved. The findings of this study provide a strong rationale for the clinical evaluation of statin therapy for the prevention and treatment of HIV-associated PAH.

## Methods

### Animals

28 adult Chinese rhesus macaques (*Macaca mulatta*) aged 6–10 years old were obtained from national primate centers or vendors and housed in accordance with the *NIH Guide for the Care and Use of Laboratory Animals*^53^ in a BSL2+ primate facility at the University of Georgia. Prior to admission to the study, all animals were screened and found negative for simian retroviruses and preexisting cardiovascular disease.

### Study design and statin treatment

All cohorts were infected with SIV/Delta B670^54,55^ (1:100 in PBS), tissue culture infectious dose of 50% (TCID_50_) = 2.6 × 10^5^), intravenously or mucosally as previously described^36,41^. To determine if statin prevention therapy in healthy macaques could alter the incidence or progression of SIV-PAH, atorvastatin treatment was initiated in a subset of NHPs 1 week prior to SIV infection (SIV/Statin Group 2; n = 14).To test the hypothesis that therapeutic treatment with atorvastatin could alter the incidence or progression of PAH after SIV-infection, treatment was initiated in a second cohort 4 months following SIV-infection (SIV/Statin Group 3; n = 14). SIV/Statin-treated cohorts received 10 mg/day atorvastatin, orally. SIV/Statin treated cohorts were compared to untreated historical controls (SIV/Untreated Group 1) previously described in Tarentelli *et al*.^36^ and Schweitzer *et al*.^41^. All procedures were approved by the University of Georgia Institutional Animal Care and Use Committee.

### Hemodynamic measurements through right heart catheterization (RHC)

Right heart catheterization (RHC) was performed and analyzed as previously described^36^ using a Swan-Ganz balloon wedge pressure catheter advanced through the right atrium, right ventricle, and pulmonary artery. Mean arterial pulmonary pressue (mPAP) is calculated from right ventricular systolic pressure (RVSP), where mPAP is reported as:

$$mPAP\,(in\,mmHg)=0.65\times (RVSP)+0.55$$

### Flow cytometry

Peripheral blood and bronchoalveolar lavage fluid (BALF) were collected at baseline (BL), 6 months post-infection (6mpi) and at study termination (10–12mpi), and processed for flow cytometry^56,57^ as previously described. CD14^dim^CD16^+^ non-classical monocytes and CD14^+^CCR7^−^CD163^−^CD206^+^ BALF macrophages were identified by flow cytometry and calculated as previously described^41^. All analyses were performed using FlowJo Analysis software (Tree Star, Inc., Ashland, OR).

### Cytokine measurement in plasma and BALF

Quantitative analysis of cytokines and chemokines in the plasma and BALF was performed using the Cytokine 29-Plex Monkey Panel (Invitrogen, Carlsbad, CA) according to the manufacturer’s instructions. BALF analytes were normalized on the assumption that plasma and BALF have equal urea concentrations as previously described^41^, using Quanti Chrom Urea Assay Kit (BioAssay Systems, Destin, FL).

### Histopathology and quantification of right ventricular and lung periarteriolar collagen deposition

5 µm thick FFPE sections of the right heart and lung were cut and stained with Masson’s trichrome and 0.1% Pico Sirius Red counterstained with Weigert’s hematoxylin, to reveal fibrillar collagen, respectively by the UGA CVM Histopoathology Laboratory (Athens, GA). Whole slide images were acquired by Servicebio (Woburn, MA). To quantify right ventricular collagen deposition in Masson’s trichrome stained sections, 170 mm^2^ regions were analyzed using Image J software (https://imagej.nih.gov/ij), with the threshold color plugin set to RGB; bright blue collagen was selected, converted to a binary image, and measured to quantify the collagen area. Collagen deposition results are reported as:

$$ \% RV\,collagen=\frac{total\,collagen\,area}{total\,muscle\,area}\times 100 \% .$$

To quantify periarteriolar collagen deposition in Picro-Sirius Red stained sections, the average of five small pulmonary vessels approximately <100 µm were analyzed using Image J software, with the color deconvolution plugin (http://www.mecourse.com/landinig/software/cdeconv/cdeconv.html) followed by application of the MRI fibrosis tool (http://dev.mri.cnrs.fr/projects/imagej-macros/wiki/Fibrosis_Tool) to quantify percentage area of fibrosis using the default settings (red 1: 0148, green 1: 0772, blue 1: 0.618, red 2: 0.462, green 2: 0.602, blue 2: 0.651, red 3: 0.187, green 3: 0.523, blue 3: 0.831)^58^. Periarteriolar collagen deposition results are reported as:

$$ \% Area\,threshold=\frac{total\,collagen\,area}{total\,vessel\,area}\times 100 \% $$

### Statistical analysis

All statistical analyses were performed using GraphPad Prism (GraphPad Software, La Jolla, CA). Continuous outcomes were summarized using mean ± SD. Serial and group characterizations of hemodynamics (mPAP, RVSP), CD4+ T cell count, and viral load, were analyzed using repeated measures mixed modeling. In each model, the main effects of group and time were included, as well as their interaction.

For hemodynamic data and CD4+ T cell count, post hoc analysis of the significant group by time interaction was performed based on Fisher’s least significant difference procedure for pairwise differences.

Following assessment of an overall significant group effect using the Kruskal-Wallis test, differences in cytokines, monocytes and macrophage phenotypes, and collagen deposition were analyzed using Mann-Whitney U tests. To test for associations between mPAP and immune markers, Spearman rank correlation was used to evaluate associations between mPAP and immune markers. Fisher’s exact test used to determine association between statin treatment and the incidence of PAH in the treated cohorts compared the untreated group while the relative risk was calculated using Koopman asymptotic score.

## Supplementary information

Supplementary Dataset 1
